# Short- and intermediate-term outcomes of transcatheter aortic valve replacement in low-risk patients: A meta-analysis and systematic review

**DOI:** 10.1016/j.ijcha.2024.101458

**Published:** 2024-07-05

**Authors:** Hammad Rahman, Priyanka Ghosh, Fahad Nasir, Muhammad A. Khan, Najeeb Rehman, Saurabh Sharma, Daniel Sporn, Edo Kaluski

**Affiliations:** a Division of Cardiology, Guthrie Robert Packer Hospital, Sayre, PA, USA; b Department of Medicine, Miami Valley Hospital, Dayton, OH, USA; c Department of Medicine, Guthrie Robert Packer Hospital, Sayre, PA, USA; d Division of Cardiology, Guthrie Health System/ Robert Packer Hospital, Sayre, PA, USA; e Division of Cardiology, Rutgers New Jersey Medical School, Newark, NJ, USA; f Division of Cardiology, The Geisinger Commonwealth Medical College, Scranton, PA, USA

**Keywords:** TAVR, SAVR, Transcatheter aortic valve replacement, Low-risk, All-cause death

## Abstract

**Background:**

Transcatheter aortic valve replacement (TAVR) being currently employed in low surgical risk patients with severe symptomatic aortic stenosis (AS). The durability and extended outcomes of TAVR as compared to surgical aortic valve replacement (SAVR) in low-risk patients remains uncertain.

**Methods:**

We selected randomized controlled trials (RCT) comparing outcomes of TAVR vs. SAVR in low surgical risk patients having severe AS using online databases. The primary outcome was all-cause death. The secondary outcomes were composite of all-cause death & disabling stroke, cardiovascular (CV) death, stroke, myocardial infarction (MI), permanent pacemaker (PPM) placement, new onset atrial fibrillation (AF), valve re-intervention and valve thrombosis. The outcomes were stratified at short- (1-year) and intermediate-term (≤5 years) follow-up. We used a random effect model to report outcomes as relative risk (RR) with a 95 % confidence interval (CI).

**Results:**

The analysis consisted of six RCTs comprising 5,122 subjects with a mean age of 75.4 years. At short-term follow up, there was a significant reduction in all-cause death (RR: 0.62, 0.46–0.82, p = 0.001) and composite of all-cause death and disabling stroke (RR: 0.62, 0.45–0.83, p = 0.002) in patients undergoing TAVR. At intermediate-term follow-up, there was no significant difference in survival (RR:0.95, 0.73–1.24, p = 0.71) and composite outcome (RR: 0.95, 0.74–1.22, p = 0.71). TAVR patients had lower incidence of new onset AF, however, higher PPM placement.

**Conclusion:**

In patients with severe AS having low-surgical risk, patients undergoing TAVR had improved short-term survival as compared to SAVR. This survival advantage was absent at intermediate-term follow-up. The long-term outcomes remain uncertain.

## 1 Introduction

Transcatheter aortic valve replacement (TAVR) is the preferred choice of treatment for most patients with severe aortic stenosis (AS) especially in those older than 75-years of age[1], [2]. The short-term outcomes of the PARTNER 3 trial (Safety and Effectiveness of the SAPIEN 3 Transcatheter Heart Valve in Low Risk Patients with Aortic Stenosis) and Evolut LOW RISK study (Evolut Surgical Replacement and Transcatheter Aortic Valve Implantation in Low Risk Patients) demonstrated improved composite of all-cause mortality, stroke and rehospitalization in low-risk patients having TAVR in comparison to surgical aortic valve replacement (SAVR)[3], [4]. This led to approval of TAVR by Food and Drug Administration (FDA) in low-risk patients, however the durability and long-term outcomes of TAVR in subjects who are expected to live longer remains uncertain. A post-hoc analysis of CoreValve US High Risk Pivotal and SURTAVI (The Surgical Replacement and Transcatheter Aortic Valve Implantation) randomized controlled trials (RCTs) comprising of 2,099 patients found lower structural valve deterioration (SVD) at 5 years in patients with TAVR as compared to SAVR employing tissue valves[5]. The study also found a strong direct association of SVD with mortality[5]. One of the pioneer studies by Waksman and colleagues, single arm trial, in low-risk patients undergoing TAVR revealed 3.3 % cardiovascular (CV) mortality and 11.9 % all-cause mortality at 4-year follow-up favoring feasibility of TAVR in low-risk cohort[6]. The NOTION (The Nordic Aortic Valve Intervention) trial is the only RCT providing long-term (10-year) outcomes, although it was a low-powered study and employed older generation TAVR valves. It comprised of 280 patients with a mean Society of Thoracic Surgery (STS) score of 3 and demonstrated no difference in all-cause mortality among the two strategies up to 10-years of follow-up[7].

Of note, the cohort of patients undergoing TAVR studied in previous trials were not exactly comparable with patients in SAVR arm. A higher proportion of patients undergoing SAVR had concomitant procedures and even redo sternotomies which increased the procedural risk compared to isolated AVR[8], [9]. Additionally, an extensive list of exclusion criteria and heterogeneity in the SAVR valves makes these trials less applicable to an average patient. These limitations hinder our ability to apply the findings of such trials in the clinical setting and necessitate reviews such as this one of large study populations to answer this question. With the recent publication of five studies evaluating outcomes of TAVR in low surgical risk patients[7], [8], [9], [10], [11], we conducted this *meta*-analysis to assess the short- and intermediate-term effects of TAVR in low-risk patients having severe AS.

## 2 Methods

The *meta*-analysis was performed in accordance with Cochrane collaboration guidelines[12] and Systematic Reviews and Meta-Analyses (PRISMA) report [13]. This study utilized data from already published trials, so the Institutional Review Boards (IRB) approval and informed consent were waived. The articles were searched using online databases (PubMed, Google scholar and CENTRAL) by two authors (H.R. & M.A.K). The search strategy and key words are alluded to in Supplementary (S) material. We reviewed citations and bibliographies of the relevant *meta*-analyses, review articles and RCTs to complete the search. The EndNote X9 (Clarivate, Philadelphia, Pennsylvania, USA) was used to upload references.

The following inclusion criteria were applied; 1) RCTs comparing balloon expandable (BEV) or self-expanding (SEV) TAVR vs. bioprosthetic SAVR 2) patients ≥ 18 years old 3) patients deemed low surgical risk having severe native AS 4) evaluation of all-cause mortality 5) ≥ 1 year follow-up. The type of surgical aortic valve was up to the discretion of the surgeon, however mechanical valves were excluded. Excluded were patients having isolated severe aortic insufficiency and severe non-aortic valve disease which could be managed by a surgical procedure. The primary outcome was all-cause death. The secondary outcomes included composite endpoint of all-cause death or disabling stroke which were extracted exactly as it was defined by each study. The remainder of secondary endpoints were CV death, stroke, myocardial infarction (MI), new onset atrial fibrillation (AF), permanent pacemaker (PPM) placement, aortic valve re-interventions and valve thrombosis (clinical and subclinical). Stroke was defined as either nondisabling or disabling and incorporated as provided by each study. All the included studies defined the clinical outcomes according to the Valve Academic Research Consortium-2 (VARC-2)[14]. We stratified the clinical outcomes based on short- (1-year) and intermediate-term (≤5-year) follow-up. In addition, analysis was performed to measure echocardiographic features including differences in aortic valve area (AVA), mean gradient and moderate or severe paravalvular regurgitation (PVR) by the end of the intermediate-term follow-up period.

Using the above-mentioned criterion, the articles were screened at the title and abstract level and then a full-text inspection performed by two authors (H.R. & P.G.) under supervision of third party (S.S., D.S & E.K.) and final articles were selected by consensus. Subsequently data collection was performed by two authors (H.R. & M.U.K.) using three forms including baseline demographics (study design, patient characteristics, type of valve, follow-up duration), echocardiographic and functional features, and outcomes of interest. The included RCTs were assessed for quality using the Cochrane bias risk assessment (H.R.) as provided in Table S1. [15].

The random-effect model was used to conduct the *meta*-analysis by authors (H.R. & M.A.K) and the estimates were reported as a risk ratio (RR) with a 95 % confidence interval (CI)[16]. We used standard difference in means (SD) to measure difference in AVA and mean gradient between the two groups. The outcome was considered statistically significant with a p-value of ≤ 0.05. Forest plots generated by the *meta*-analysis software were used to illustrate the results. We included events reported by the studies for each outcome up-to 1-year (short-term) and 5-year (intermediate-term) follow-up to calculate the relative risk. Only the NOTION trial provided outcomes beyond five years, however it consisted of minor portion of the total included participants in this *meta*-analysis, so we decided to include NOTION trial outcomes at 5-year follow-up[7], [17]. The Q statistics was utilized to measure heterogeneity and calculated with the *I^2^* index [18]. The sensitivity analysis was performed to evaluate the robustness of data. Moment of method random-effect *meta*-regression analysis was conducted for the primary outcome to assess association of results with baseline covariates. Comprehensive Meta-analysis software version 4.0 (Biostat, Englewood, NJ) was used to run the *meta*-analyses.

## 3 Results

Six RCTs comprising 5,122 subjects were selected following the above inclusion criteria[8], [9], [10], [11], [17], [19] although the data was collected from 10 publications of included RCTs at various durations of follow-up[8], [9], [10], [11], [17], [19], [20], [21], [22], [23]. The detailed search strategy is provided in the supplementary figure S1. All the included trials were multicentered, three studies were inter-continental[8], [9], [11], and three studies were conducted in Europe only[10], [17], [19]. Two studies provided outcomes up to 1-year[10], [19] and rest of the studies extended follow-up beyond 1-year[8], [9], [11], [17]. The PARTNER 3 trial utilized the SAPIEN 3 BEV in severe AS patients with a mean STS score of 1.9 and reported outcomes up to five years[8]. The Evolut LOW RISK study enrolled severe AS patients with a mean STS score of 1.95 and reported outcomes up to four years[9]. The TAVR valves used in this trial were the SEV CoreValve (3.6 %), Evolut R (74.1 %), and Evolut PRO (22.3 %)[23]. The NOTION trial enrolled subjects with severe AS regardless of predicted risk score for death[21]. The mean STS score of the included patients was 3.0 and > 80 % of patients were considered low risk. The self-expandable CoreValve was deployed for the TAVR patients by transfemoral or subclavian approach. The UK TAVI (The UK Transcatheter Aortic Valve Implantation) study enrolled ≥ 70-year-old subjects with severe symptomatic AS with mean STS score of 2.6 % and > 80 % of patients with STS < 4 %[19]. The TAVR valve could be either SEV or BEV.

The DEDICATE-DZHK6 (Transcatheter or Surgical Aortic Valve Replacement) and VIVA (Transcatheter Aortic Valve Replacement Versus Surgical Aortic Valve Replacement for Treating Elderly Patients with Severe Aortic Stenosis and Small Aortic Annuli) trials recruited subjects ≥ 65-year-old at low to intermediate surgical risk with severe AS and randomized to TAVR (BEV or SEV) vs. SAVR[10], [11]. Of note, VIVA trial included patients with small aortic annulus with a mean 23 mm diameter and 93 % were women[11]. The STS score was < 4 % in more than 90 % among subjects in both DEDICATE-DZHK6 and VIVA trials[10], [11]. The detailed baseline characteristics of the included patients are provided in Table 1 and procedural characteristics in Table S2. Among the SAVR arm in this study, 18.9 % of patients underwent concomitant procedures which is much higher than the TAVR patients. The SAVR arm in the latest study, DEDICATE-DZHK6, had one of the lowest 4.3 % concomitant procedures[10]. Most patients in the SAVR arm underwent full sternotomy although only three studies provided sternotomy approach details[8], [10], [19]. The participants of this study had a mean AVA of 0.79 cm2, mean gradient of 46.3 mmHg with a mean age of 75.4 years. We provided clinical outcomes of interest extracted from each study in Tables S3 and S4. The echocardiographic and functional features at baseline and by the end of the intermediate-term studies furnished in a separate Table S5.

**Table 1:** Baseline characteristics of included randomized controlled trials.

**Studies**	**Intervention group (number)**	**Design**	**Sites**	**Age (years)**	**Female (%)**	**Mean STS score (%)**	**Prior MI (%)**	**Prior stroke (%)**	**NYHA III/IV symptoms**	**AF (%)**	**AVA (cm2)**	**Mean Gradient (mmHg)**	**Type of TAVR device**	**Follow-up duration (months)**
**DEDICATE-DZHK6 (2024)**[10]	TAVR (7 0 1)	MCT	Germany	74.3	44	1.8	5.2	6.1	46.2	28.9	0.8	46.5	Balloon expandable (61.4 %), self-expanding (35.1 %)	12
	SAVR (7 1 3)			74.6	42.7	1.9	7.5	6.0	45.6	27.4	0.8	45.0		
**VIVA (2024)**[11]	TAVR (77)	MCT	Canada, Europe & Brazil	75.9	94.8	2.55	−	−	29.9	7.8	0.67	47.0	Balloon expandable (40.8 %), self-expanding (59.2 %)	24
	SAVR (74)			75.1	90.5	2.43	−	−	32.4	18.9	0.74	49.0		
**PARTNER 3 (2023)**[8]	TAVR (4 9 6)	MCT	USA, Austrailia, New Zealand	73.3	32.5	1.9	5.7	3.4	31.2	15.7	0.8	49.4	Balloon expandable SAPIEN 3 valve system	60
	SAVR (4 5 4)			73.6	28.9	1.9	5.8	5.1	23.8	18.8	0.8	48.3		
**EVOLUT Low Risk (2023)**[9]	TAVR (7 3 0)	MCT	Northern America, Europe, Japan, Australia, New Zealand	74.1	36.4	2.0	6.7	10.1	24.9	15.4	0.8	47.0	Self-expanding CoreValve (3.6 %), Evolut R (74.1 %), Evolut PRO (22.3 %)	48
	SAVR (6 8 4)			73.7	34.1	1.9	4.8	12.0	28.2	14.4	0.8	46.6		
**UK TAVI (2022)**[19]	TAVR (4 5 8)	MCT	United Kingdom	81.0	46.1	2.6	9.5	5.7	40.3	24.0	0.7	43.1	Balloon expandable (57.3 %), self-expanding (29.8 %)	12
	SAVR (4 5 5)			81.0	46.8	2.7	8.9	5.1	45.2	24.3	0.7	44.0		
**NOTION (2019)**[17]	TAVR (1 4 5)	MCT MCT	Denmark, Sweden	79.2	46.2	2.9	5.5	16.6	48.6	27.8	0.7	43.4	Self-expanding CoreValve bioprosthesis	60
	SAVR (1 3 5)			79.0	47.4	3.1	4.4	16.3	45.5	25.6	0.7	44.9		

## 4 Short-term (1-year) outcomes

**Fig. 1:**
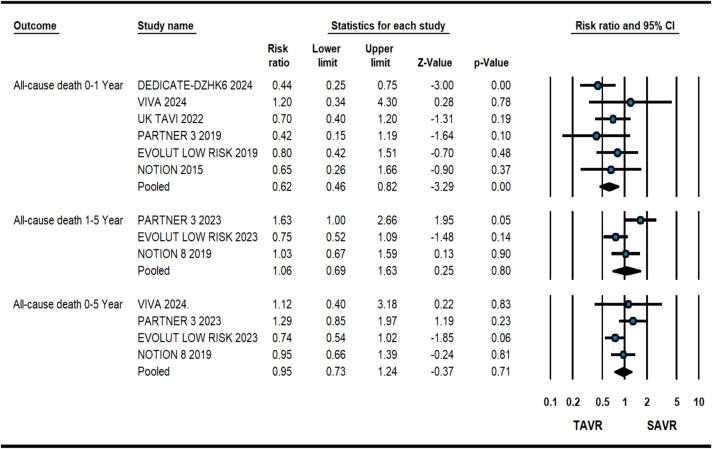
Forest plot comparing transcatheter aortic valve replacement (TAVR) vs. surgical aortic valve replacement (SAVR) for all-cause death.

**Fig. 2:**
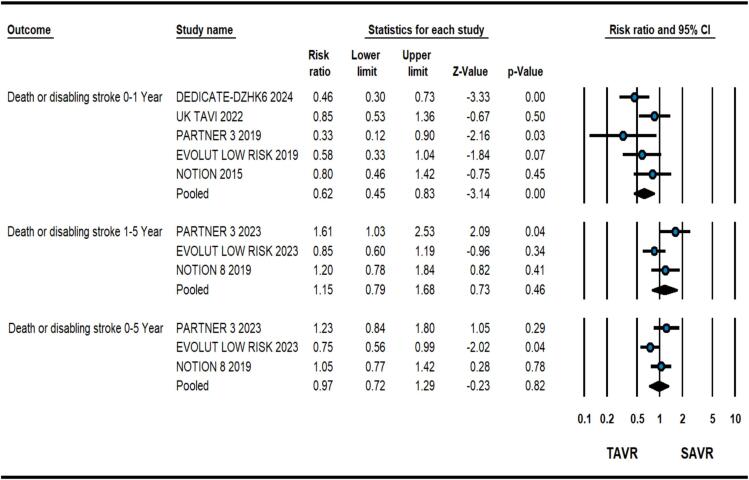
Forest plot comparing transcatheter aortic valve replacement (TAVR) vs. surgical aortic valve replacement (SAVR) for composite of all-cause death and disabling stroke.

**Fig. 3:**
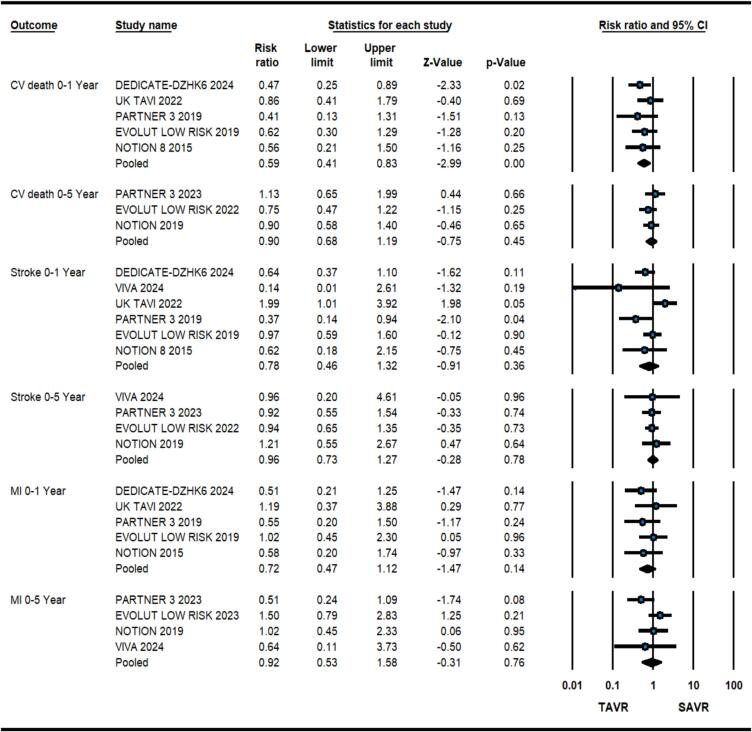
Forest plot comparing transcatheter aortic valve replacement (TAVR) vs. surgical aortic valve replacement (SAVR) for cardiovascular (CV) death, stroke and myocardial infarction (MI).

At 1-year, patients having severe AS at low surgical risk receiving TAVR had 38 % relative risk reduction of all-cause death in comparison to SAVR (2.8 % vs. 4.6 %, RR: 0.62, 0.46–0.82, p = 0.001, *I*^2^ = 0) as illustrated in Fig. 1. Meta-regression analysis did not reveal any significant association of baseline variables with the primary outcome. Similarly, there was a significant reduction in composite of all-cause death and disabling stroke (3.9 % vs. 6.4 %, RR: 0.62, 0.45–0.83, p = 0.002, *I*^2^ = 30) with TAVR (Fig. 2). TAVR strategy also led to improved CV death (1.9 % vs. 3.4 %, RR: 0.59, 0.41–0.83, p = 0.001, *I*^2^ = 0), however, no difference was found in stroke (3.2 % vs. 3.8 %, RR: 0.78, 0.45–1.33, p = 0.36, *I*^2^ = 59) and MI (1.4 % vs. 1.97 %, RR:0.72, 0.47–1.12, p = 0.14, *I*^2^ = 0) (Fig. 3). As expected, TAVR patients had lower incidence of new-onset atrial fibrillation (10.5 % vs. 35.3 %, RR:0.29, 0.21–0.41, p < 0.001, *I*^2^ = 82) but higher need for PPM placement (14.9 % vs. 6.3 %, RR:2.40, 1.54–3.72, p < 0.001, *I*^2^ = 80) (Figure S2). No significant difference was observed in valve re-intervention (0.9 % vs. 0.5 %, RR: 1.63, 0.82–3.24, p = 016, *I*^2^ = 0) and valve thrombosis (0.6 % vs. 0.3 %, RR:2.04 %, 0.64–6.63, p = 0.23, *I*^2^ = 2.9) (Figure S3).

## 5 Intermediate-term (0–5 year) outcomes

At a mean follow-up duration of 4.3 years including outcomes from four studies, there was no significant difference in all-cause death in patients having TAVR vs. SAVR (11.3 % vs. 12.2 %, RR:0.95, 0.73–1.24, p = 0.71, *I*^2^ = 32) as shown in Fig. 1. Upon calculation of 1–5-year primary outcome, there was no significant difference in all-cause death (RR: 1.06, 0.69-0.163, p = 0.82, *I*^2^ = 66) although patients with BEV (PARTNER-3 trial) had higher mortality during 1–5 years follow-up (Fig. 1)[8]. The two procedural strategies had no significant differences in composite of all-cause death or disabling stroke from 0 to 5 Year (13.7 % vs. 14.7 %, RR: 0.95, 0.74–1.22, p = 0.71, *I*^2^ = 38) and 1–5 Year (RR: 1.15, 0.79–1.68, p = 0.46, *I*^2^ = 61) follow-up (Fig. 2).

Among the secondary outcomes, there were no significant differences regarding CV death (6.2 % vs. 6.9 %, RR:0.90, 0.68–1.19, p = 0.45, *I*^2^ = 0), stroke (6.6 % vs. 6.9 %, RR:0.96, 0.73–1.27, p = 0.78, *I*^2^ = 0), MI (3.2 % vs. 3.4 %, RR:0.92, 0.53–1.58, p = 0.76, *I*^2^ = 37) (Fig. 3.), valve re-intervention (1.8 % vs. 1.9 %, RR:0.97, 0.0.56–1.69, p = 0.91, *I*^2^ = 0) and valve thrombosis (1.3 % vs. 0.4 %, RR:3.03, 0.32–28.38, p = 0.33, *I*^2^ = 71) (Figure. S3). There was a significantly reduced relative risk of new onset atrial fibrillation with TAVR vs. SAVR (13.2 % vs. 39.4 %, RR:0.34, 0.29–0.39, p < 0.001, *I*^2^ = 0) (Fig. 3). Contrarily, TAVR led to increased need for PPM placement as compared to SAVR (21.4 % vs. 9.4 %, RR:2.40, 1.40–4.12, p < 0.001, *I*^2^ = 80) (Figure. S2).

Among the echocardiographic features, TAVR group had significantly better AVA (cm2) by the end of the follow-up period (SD:0.25, 0.14–0.36, p < 0.001, *I*^2^ = 17) (Figure S4). The difference was mainly observed in patients with SEV[9], [17]. Similarly, there was significantly lower mean pressure gradient in patients with SEV[9], [17]. Conversely, there was significantly higher moderate or severe PVR in patients with TAVR (4.1 % vs. 0 %, RR: 15.3, 2.9–81.0, P = 0.001, *I*^2^ = 0) (Figure S5).

## 6 Discussion

This analysis consisting of six RCTs evaluating TAVR vs. SAVR in low surgical risk patients having severe AS revealed 38 % relative risk reduction with TAVR in all-cause death as well as composite of all-cause death and disabling stroke at 1-year post index procedure. There was a significant reduction in CV death among TAVR patients at short-term follow-up. The benefits observed with TAVR regarding short-term survival, composite outcome and CV death were lacking at intermediate-term follow up with no difference between the two strategies. The study found no differences in stroke, MI, valve thrombosis and valve re-interventions. As expected, there was a significantly higher need for PPM placement (21.4 % vs. 9.4 %) in TAVR patients. The diagnosis of new onset of AF was significantly lower in patients getting TAVR (13.2 % vs. 39.4 %). At intermediate-term follow-up, TAVR patients had better mean AVA with higher risk of moderate or severe PVR.

The long-term results of previous *meta*-analyses and RCTs showed promising results for TAVR in comparison to surgery in patients having severe AS at intermediate to high risk[1], [2], [24], [25], [26]. The patients at low-surgical risk are usually expected to live longer and the extended effects of TAVR had been uncertain in this cohort. Ahmed et al, a *meta*-analysis comprising of four RCTs[27], demonstrated significantly reduced all-cause mortality as well as composite of mortality and disabling stroke at one year of follow-up in TAVR vs. SAVR patients in low-risk patients. The benefit was not found at > 1 year follow up, however the analysis consisted predominantly of outcomes up to 2-year. A separate *meta*-analysis, Sȧ et al, reporting mid-term outcomes of TAVR in low-risk patients, found improved survival after 2 years with SAVR based on propensity-score-matched observational studies, however similar findings were not established on randomized data[28]. Both above-mentioned *meta*-analyses[27], [28] did not include 1-year outcomes of VIVA[11] and DEDICATE-DZHK6 trials[10] as well as four-year and five-year results of the Evolut LOW RISK and the PARTNER 3 respectively[8], [9]. Our analysis comprising of the updated results, showed benefit of TAVR regarding all-cause death (p = 0.001) and composite of all-cause death & stroke (p = 0.002) limited to 1-year follow-up. The advantage of TAVR during the 0–1 Year outcomes is likely related to higher intraprocedural and early postoperative morbidity and mortality in SAVR patients. The year 1–5 and year 0–5 analyses revealed no difference in the all-cause death and composite endpoint among subjects receiving TAVR vs. SAVR. The long-term results (5–10 Year) of future trials would provide more insight regarding the durability of TAVR valves in low-risk patients.

Among the intermediate follow-up studies, the risk of all-cause mortality at year-4 was lower in the Evolut LOW RISK TAVR group (6.3 %) compared to the SAVR (12.4 %), although it was not statistically significant[9]. The trial utilized SEV Evolut R and Evolut PRO aortic valves. There is no detail mentioned in the Evolut LOW RISK study regarding the type of bioprosthetic valve in the SAVR group and sternotomy approach[23]. In the PARTNER 3 study which studied the BEV SAPIEN 3 valve, the rate of death at year-4 was slightly higher in the TAVR arm (7.4 %) than the SAVR group (5.9 %)[8]. There was a non-favorable trend in all-cause mortality with BEV in the PARTNER3 trial during the year 1–5 follow-up[8]. This difference might be related to reasons including higher loss to follow-up in the SAVR arm of 11.9 % in the PARTNER3 trial, 36 % concomitant procedures (12.8 % coronary artery bypass grafting) in the SAVR group leading to improved 1–5 Year surgical outcomes and the hemodynamic effects and longevity of BEV itself[8]. The 5-year results of CHOICE trial showed no significant difference in clinical outcomes among the older generation SEV and BEV except slightly higher need for PPM with SEV. There was significantly better mean effective orifice area, mean *trans*-aortic gradients and end-diastolic left ventricular dimensions with SEV[29]. Similarly, a metanalysis by Wang et al comprising of 4 RCTs and 14 prospective score-matched studies revealed significantly better mean AVA and mean gradient in patients receiving SEV compared to BEV, although higher rate of PVR with SEV[30]. VARC-3 technical success rate is usually > 85 % for both SEV and BEV valve types[31], [32].

The NOTION trial recruited participants in the early 2010′s and investigated TAVR with the self-expandable CoreValve[21]. Almost 81 % of participants were considered low risk based on the STS score. At 5, 8 and 10 years of follow-up, there was similar risk of composite endpoint with TAVR and SAVR[7]. The overall rate of composite outcome in the NOTION study was higher than the Evolut LOW RISK and PARTNER 3 trials. This is likely related to a higher mean STS score (3.0) in the NOTION study participants and non-contemporary valve technology and medical therapy. At 10 years, bioprosthetic valve failure (composite of valve related death, valve reintervention and severe structural valve deterioration) occurred in 10.8 % of the TAVR and 15.1 % of the SAVR group with no significant difference indicating durability of TAVR in the low-risk population[7].

The percentage of patients younger than 65 years of age had been quite small in the low-risk TAVR trials[7], [8], [9], [10], [11], [19]. There is lack of evidence regarding the durability of the TAVR in a younger population with less comorbidities. In younger adults who are supposed to survive longer after TAVR, there are several concerns which need answers, including but not limited to: impact of complex coronary artery disease, feasibility and ease of revascularization, conduction system complications, subclinical valve thrombosis and paravalvular regurgitation[33]. Ongoing studies like EARLY-TAVR will provide more insight into TAVR outcomes in asymptomatic severe aortic stenosis patients[34].

There are several limitations of this study. First, there is heterogeneity in the baseline population, need for concomitant procedures, kind of TAVR valves and SAVR valves used in the six trials[8], [20], [35]. Second, the NOTION trial enrolled all-comer participants with a mean STS score of 3 % higher than rest of the studies, although > 81 % of patients were low surgical risk; hence, we included the study in our analysis[21]. In our study, we included a composite of all-cause death and disabling stroke, the NOTION study instead provided a composite result of all-cause mortality, stroke, and myocardial infarction[17]. The VIVA trial recruited majority female subjects with small aortic annulus which might have increased heterogeneity in outcomes[11]. The results of this *meta*-analysis may not apply to young adults with severe aortic stenosis, and isolated severe aortic insufficiency. Additionally, there was variation in loss to follow up among the TAVR and SAVR groups leading to potential bias in the results[8]. One of the major limitations of the intermediate-term outcomes is that it reflects a considerable outdated TAVR practice: since practically most of TAVR valves studied do not reflect the contemporary valve designs which are far superior to the 1st (CoreValve) 2nd (Evolut R and Pro) or 3rd (S3) generation valves and delivery systems. Similar limitations also apply to SAVR technology and valves which are constantly being refined and improved. We also could not run analysis on structural valve deterioration outcomes due to lack of long-term data.

Moreover, the TAVR operator nowadays has the choice of various excellent valve designs tailored to the patient’s anatomy or clinical scenario to provide optimal outcomes along with the best hemodynamics while avoiding prosthetic mismatch and procedural complications. This is especially relevant to subjects with small annulus size. The study did not analyze other parameters of these procedures including length of intensive care and hospital stay, duration of post-procedural functional disability and unemployment which would clearly favor TAVR and other surgical-related complications like chest wall and wound complications. Finally, there are certain patient categories that were clearly omitted from these studies including bicuspid valves, or subjects with multiple or specific co-morbidities deeming them “not suitable for SAVR”.

In conclusion, low-surgical risk patients with severe aortic stenosis who are suitable for both TAVR and SAVR, TAVR had improved survival at 1-year compared to SAVR, however, this advantage was not extended to intermediate-term survival. The SAVR group increased initial mortality is due to a more invasive surgery (sternotomy, cardiac arrest, and cardiopulmonary bypass), although this gap in mortality narrows over time to become non-significant which may indicate higher rates of mortality (related to increased rates of PPM insertion and moderate-severe PVR) in the TAVR group after year-1 of index procedure. The durability and practicability of the TAVR valve in younger low risk patients remains uncertain and requires further investigation.

## 7 Declaration of generative AI in the writing process

The authors (H.R & M.A.K) utilized Comprehensive Meta-analysis software version 4.0 (Biostat, Englewood, NJ) to run the *meta*-analysis. After using this tool, the authors reviewed and edited the content as needed and take full responsibility for the content of the publication.

## Ethical statement

This study is exempt from ethics approval as the data is based on previously published trials where informed consent was obtained by investigators.

## CRediT authorship contribution statement

**Hammad Rahman:** Writing – review & editing, Writing – original draft, Visualization, Validation, Supervision, Software, Resources, Project administration, Methodology, Investigation, Formal analysis, Data curation, Conceptualization. **Priyanka Ghosh:** Writing – original draft, Visualization, Validation, Methodology. **Fahad Nasir:** Validation, Project administration, Methodology, Data curation. **Muhammad A. Khan:** Visualization, Validation, Methodology, Formal analysis, Data curation. **Najeeb Rehman:** Validation, Supervision. **Saurabh Sharma:** Writing – review & editing, Validation, Supervision, Methodology. **Daniel Sporn:** Writing – review & editing, Supervision, Conceptualization. **Edo Kaluski:** Writing – review & editing, Validation, Supervision, Project administration, Methodology, Investigation, Conceptualization.

## Declaration of competing interest

The authors declare that they have no known competing financial interests or personal relationships that could have appeared to influence the work reported in this paper.
