# Supplementary material for: Short- and intermediate-term outcomes of transcatheter aortic valve replacement in low-risk patients: A meta-analysis and systematic review
Source: Int J Cardiol Heart Vasc. 2024 Jul 5;53:101458. doi: 10.1016/j.ijcha.2024.101458 (PMC11265583; doi:10.1016/j.ijcha.2024.101458)

**Short- and Intermediate-Term Outcomes of Transcatheter Aortic Valve Replacement in Low-Risk Patients: A Meta-Analysis and Systematic Review**

Hammad Rahman, MD; Priyanka Ghosh, DO; Fahad Nasir, MD; Muhammad A. Khan, MD; MD; Najeeb Rehman, MD, FACC; Saurabh Sharma, MD, FACC; Daniel Sporn MD, FACC; Edo Kaluski, MD, FACC

**Contents**

**Search Strategy and PRISMA flow diagram, figure S1**-------------------------------------------------------------------------Page 1

**Table S1:** Cochrane quality assessment tool for assessment of risk of bias for the randomized trials -----------------------Page 2

**Table S2**: Procedural characteristics, LOS, length of stay; SAVR, surgical aortic valve replacement; TAVR, transcatheter aortic valve replacement-----------------------------------------------------------------------------------------------------------------Page 3

**Table S3**: Clinical outcomes of interest provided in each study at 1-year follow up -------------------------------------------Page 4

**Table S4**: Clinical outcomes of interest provided in each study up to 5-year follow up ---------------------------------------Page 5

**Table S5**: Echocardiographic and functional characteristics at baseline and end of the studies ------------------------------Page 6

**Figure S2:** Forest plot comparing transcatheter aortic valve replacement (TAVR) vs. surgical aortic valve replacement (SAVR) for new onset atrial fibrillation and permanent pacemaker (PPM) placement -----------------------------Page 7

**Figure S3:** Forest plot comparing transcatheter aortic valve replacement (TAVR) vs. surgical aortic valve replacement (SAVR) for aortic valve re-intervention and valve thrombosis----------------------------------------------------------Page 8

**Figure S4**: Forest plot comparing transcatheter aortic valve replacement (TAVR) vs. surgical aortic valve replacement (SAVR) for aortic valve area (cm2) and mean gradient (mmHg) by the end of study period---------------------- Page 9

**Figure S5:** Forest plot comparing transcatheter aortic valve replacement (TAVR) vs. surgical aortic valve replacement (SAVR) for moderate or severe paravalvular aortic regurgitation (PVR) by the end of study period -----------Page 10

**Key words for search strategy:**

"Transcatheter aortic valve replacement"[All Fields] OR "TAVR"[All Fields] OR "TAVI"[All Fields] OR "surgical aortic valve replacement"[All Fields] OR "SAVR"[All Fields] OR "SAVI"[All Fields] OR "low surgical risk"[All Fields] OR "low risk"[All Fields] OR "randomized controlled trials"[All Fields] OR "meta-analysis"[All Fields]


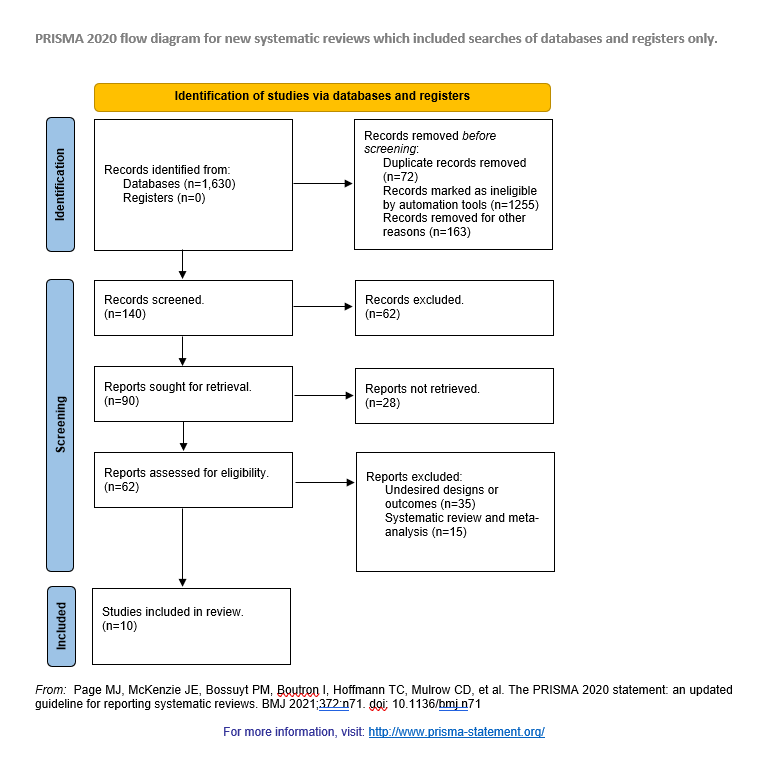


**Figure S1,** PRISMA flow diagram

| **Studies** | **Randomization** | **Allocation concealment** | **Blinding** | **Deviation from intended intervention** | **Outcome assessment bias** | **Free of other biases** |
| --- | --- | --- | --- | --- | --- | --- |
| **DEDICATE-DZHK6 (2024)** | Low risk | Low risk | High risk | Low risk | Low risk | Low risk |
| **VIVA (2024)** | Low risk | Low risk | High risk | Low risk | Unclear risk | Unclear risk |
| **PARTNER 3 (2023)** | Low risk | Low risk | High risk | Low risk | Unclear risk | Low risk |
| **Evolut LOW RISK (2023)** | Low risk | Low risk | High risk | Low risk | Unclear risk | Low risk |
| **UK-TAVI (2022)** | Low risk | Low risk | High risk | Low risk | Low risk | Unclear risk |
| **NOTION (2019)** | Unclear risk | Low risk | High risk | Low risk | Unclear risk | Unclear risk |

**Table S1:** Cochrane quality assessment tool for assessment of risk of bias for the randomized trials

| **Studies** | **Intervention Arm (number of subjects)** | **SAVR valve types** | **Access/Approach** | **Concomitant procedures (%)** | **Conscious sedation (%)** | **Median index LOS (days)** | **Discharge home (%)** | **In-hospital mortality (%)** |
| --- | --- | --- | --- | --- | --- | --- | --- | --- |
| **DEDICATE-DZHK6 (2024)** | TAVR (701) | Balloon expandable (61.4%), self-expanding (35.1%) | Transfemoral (97.3%), Transapical (2.0), Transaxillary (0.3%) | 0 | 75.1 | 5.0 | 74.7 | 0.4 |
|  | SAVR (713) | Stented (77.4%), Sutureless (15.8%), unknown (6.6%) | Sternotomy (50.9%), partial sternotomy (38.7%), other (10.4%) | 4.3 | 0 | 9.0 | 40.4 | 1.4 |
| **VIVA (2024)** | TAVR (77) | Balloon expandable (40.8%), self-expanding (59.2%) | Transfemoral (90.8%), Transcarotid/transaortic (9.2%) | 12.8 | - | 3.0 | - | - |
|  | SAVR (74) | Stented (78.9%), sutureless (21.1%) | - | 6.8 | - | 8.0 | - | - |
| **PARTNER 3 (2023)** | TAVR (496) | Balloon expandable SAPIEN 3 valve system | Percutaneous (98.9%), surgical cutdown (1.0%) | 7.9 | 65.1 | 3.0 | 95.8 | 0.4 |
|  | SAVR (454) | Edwards Lifesciences (72.2%), Medtronic (7.3%), St Jude (16.8%) | Sternotomy (74.2%), ministernotomy or thoracotomy (24.3%) | 36.1 | 0 | 7.0 | 73.1 | 0.9 |
| **EVOLUT Low Risk (2023)** | TAVR (730) | Self-expanding CoreValve (3.6%), Evolut R (74.1%), Evolut PRO (22.3%) | Transfemoral (99.0%), subclavian (0.6%), direct aortic (0.4%) | 6.9 | 43.1 | - | - | - |
|  | SAVR (684) | - | - | 26.3 | 0 | - | - | - |
| **UK TAVI (2022)** | TAVR (458) | Balloon expandable (57.3%), self-expanding (29.8%) | Transfemoral (92%), subclavian (2.2%), transapical or direct aortic (5.8%) | 7.3 | 69.6 | 3 | 94.2 | - |
|  | SAVR (455) | Stented (98.1%), Stentless (0.7%) | Midline Sternotomy (89.5%), minimally invasive (10.5%) | 21.5 | 0 | 8 | 82.6 | - |
| **NOTION 2019** | TAVR (145) | Self-expanding CoreValve bioprosthesis | Transfemoral (96.5%), subclavian (3.5%), | - | 18.3 | 8.9 | - | - |
|  | SAVR (135) | - | - | 0.7 | 0 | 12.9 | - | - |

Table S2, Procedural characteristics, LOS, length of stay; SAVR, surgical aortic valve replacement; TAVR, transcatheter aortic valve replacement.

| **Studies** | **TAVR** | | **SAVR** | |
| --- | --- | --- | --- | --- |
|  | **Events** | **Total** | **Events** | **Total** |
| **All-cause death at 1 year** | | | | |
| DEDICATE-DZHK6 2024 | 18 | 701 | 42 | 713 |
| VIVA 2024 | 5 | 77 | 4 | 74 |
| UK TAVI 2022 | 21 | 458 | 30 | 455 |
| PARTNER 3 2019 | 5 | 496 | 11 | 454 |
| EVOLUT LOW RISK 2019 | 17 | 730 | 20 | 684 |
| NOTION 2015 | 7 | 145 | 10 | 135 |
| **All-cause death or disabling stroke at 1 year** | | | | |
| DEDICATE-DZHK6 2024 | 26 | 701 | 57 | 713 |
| UK TAVI 2022 | 30 | 458 | 35 | 455 |
| PARTNER 3 2019 | 5 | 496 | 14 | 454 |
| EVOLUT LOW RISK 2019 | 18 | 730 | 29 | 684 |
| NOTION 2015 | 19 | 145 | 22 | 135 |
| **Cardiovascular death at 1 year** | | | | |
| DEDICATE-DZHK6 2024 | 14 | 701 | 30 | 713 |
| UK TAVI 2022 | 13 | 458 | 15 | 455 |
| PARTNER 3 2019 | 4 | 496 | 9 | 454 |
| EVOLUT LOW RISK 2019 | 12 | 730 | 18 | 684 |
| NOTION 8 2015 | 6 | 145 | 10 | 135 |
| **Stroke at 1 year** | | | | |
| DEDICATE-DZHK6 2024 | 20 | 701 | 32 | 713 |
| VIVA 2024 | 0 | 77 | 3 | 74 |
| UK TAVI 2022 | 24 | 458 | 12 | 455 |
| PARTNER 3 2019 | 6 | 496 | 15 | 454 |
| EVOLUT LOW RISK 2019 | 30 | 730 | 29 | 684 |
| NOTION 8 2015 | 4 | 145 | 6 | 135 |
| **Myocardial infarction at 1 year** | | | | |
| DEDICATE-DZHK6 2024 | 7 | 701 | 14 | 713 |
| UK TAVI 2022 | 6 | 458 | 5 | 455 |
| PARTNER 3 2019 | 6 | 496 | 10 | 454 |
| EVOLUT LOW RISK 2019 | 12 | 730 | 11 | 684 |
| NOTION 2015 | 5 | 145 | 8 | 135 |
| **Aortic valve re-intervention at 1 year** | | | | |
| DEDICATE-DZHK6 2024 | 4 | 701 | 2 | 713 |
| UK TAVI 2022 | 10 | 458 | 5 | 455 |
| PARTNER 3 2019 | 3 | 496 | 2 | 454 |
| EVOLUT LOW RISK 2019 | 5 | 730 | 4 | 684 |
| NOTION 8 2015 | 0 | 145 | 0 | 135 |
| **Valve thrombosis at 1 year** | | | | |
| DEDICATE-DZHK6 2024 | 5 | 701 | 2 | 713 |
| PARTNER 3 2019 | 5 | 496 | 1 | 454 |
| EVOLUT LOW RISK 2019 | 1 | 730 | 2 | 684 |
| **Permanent pacemaker placement at 1 year** | | | | |
| DEDICATE-DZHK6 2024 | 82 | 701 | 47 | 713 |
| UK TAVI 2022 | 65 | 458 | 33 | 455 |
| PARTNER 3 2019 | 38 | 496 | 25 | 454 |
| EVOLUT LOW RISK 2019 | 142 | 730 | 46 | 684 |
| NOTION 8 2015 | 51 | 145 | 3 | 135 |
| **New onset atrial fibrillation at 1 year** | | | | |
| DEDICATE-DZHK6 2024 | 86 | 701 | 211 | 713 |
| PARTNER 3 2019 | 30 | 496 | 150 | 454 |
| EVOLUT LOW RISK 2019 | 72 | 730 | 262 | 684 |
| NOTION 8 2015 | 30 | 145 | 79 | 135 |

**Table S3**: Clinical outcomes of interest provided in each study at 1-year follow up, TAVR, transcatheter aortic valve replacement; SAVR, surgical aortic valve replacement.

| **Studies** | **TAVR** | | **SAVR** | |
| --- | --- | --- | --- | --- |
|  | **Events** | **Total** | **Events** | **Total** |
| **All-cause death 0-5 Year** | | | | |
| VIVA 2024 Two-Year | 7 | 77 | 6 | 74 |
| PARTNER 3 2023 Five-Year | 48 | 496 | 34 | 454 |
| EVOLUT LOW RISK 2023 Four-Year | 64 | 691 | 76 | 610 |
| NOTION 2019 Five-Year | 40 | 145 | 39 | 135 |
| **All -cause death 1-5 Year** | | | | |
| PARTNER 3 2023 1-5 Year | 43 | 490 | 23 | 427 |
| EVOLUT LOW RISK 2023 1-5 Year | 47 | 709 | 56 | 637 |
| NOTION 8 2019 1-5 Year | 33 | 136 | 29 | 123 |
| **All-cause death or disabling stroke 0-5 Year** | | | | |
| VIVA 2024 | 7 | 77 | 8 | 74 |
| PARTNER 3 2023 Five-Year | 55 | 496 | 41 | 454 |
| EVOLUT LOW RISK 2023 Four-Year | 76 | 691 | 90 | 610 |
| NOTION 2019 Five-Year | 55 | 145 | 49 | 135 |
| **All-cause death or disabling stroke 1-5 Year** | | | | |
| PARTNER 3 2023 1-5 Year | 50 | 490 | 27 | 427 |
| EVOLUT LOW RISK 2023 1-5 Year | 58 | 706 | 61 | 628 |
| NOTION 8 2019 1-5 Year | 36 | 126 | 27 | 113 |
| **Cardiovascular death 0-5 Year** | | | | |
| PARTNER 3 2023 Five-Year | 26 | 496 | 21 | 454 |
| EVOLUT LOW RISK 2022 Three-Year | 29 | 730 | 36 | 684 |
| NOTION 2019 Five-Year | 30 | 145 | 31 | 135 |
| **Stroke 0-5 Year** | | | | |
| VIVA 2024 Two-Year | 3 | 77 | 3 | 74 |
| PARTNER 3 2023 Five-Year | 27 | 496 | 27 | 454 |
| EVOLUT LOW RISK 2023 Three-Year | 53 | 730 | 53 | 684 |
| NOTION 2019 Five-Year | 13 | 145 | 10 | 135 |
| **Myocardial infarction 0-5 Year** | | | | |
| PARTNER 3 2023 | 10 | 496 | 18 | 454 |
| EVOLUT LOW RISK 2023 | 24 | 730 | 15 | 684 |
| NOTION 2019 | 11 | 145 | 10 | 135 |
| VIVA 2024 | 2 | 77 | 3 | 74 |
| **Aortic valve re-intervention 0-5 Year** | | | | |
| VIVA 2024 Two Year | 2 | 77 | 1 | 74 |
| PARTNER 3 2023 Five-Year | 12 | 496 | 12 | 454 |
| EVOLUT LOW RISK 2023 Four-Year | 9 | 691 | 10 | 610 |
| NOTION 2019 Five-Year | 3 | 139 | 1 | 135 |
| **Valve thrombosis 0-5 Year** | | | | |
| PARTNER 3 2023 | 12 | 496 | 1 | 454 |
| EVOLUT LOW RISK 2023 | 5 | 691 | 4 | 610 |
| NOTION 2019 | 0 | 145 | 0 | 135 |
| **Permanent pacemaker placement 0-5 Year** | | | | |
| VIVA 2024 | 11 | 77 | 5 | 74 |
| PARTNER 3 2023 Five-Year | 63 | 496 | 43 | 454 |
| EVOLUT LOW RISK 2023 Four-Year | 170 | 691 | 62 | 610 |
| NOTION 2019 Five-Year | 58 | 145 | 10 | 135 |
| **New onset atrial fibrillation 0-5 Year** | | | | |
| VIVA 2024 | 8 | 77 | 23 | 74 |
| PARTNER 3 2023 Five-Year | 55 | 496 | 155 | 454 |
| EVOLUT LOW RISK 2023 Three-Year | 94 | 730 | 271 | 684 |
| NOTION 2019 Five-Year | 34 | 145 | 82 | 135 |

**Table S4**: Clinical outcomes of interest provided in each study up to 5-year follow up, TAVR, transcatheter aortic valve replacement; SAVR, surgical aortic valve replacement.

| **Studies** | | **VIVA (2024)** | | **PARTNER 3 (2023)** | | **EVOLUT Low Risk (2023)** | | **NOTION (2019)** | |
| --- | --- | --- | --- | --- | --- | --- | --- | --- | --- |
|  | | **TAVR** | **SAVR** | **TAVR** | **SAVR** | **TAVR** | **SAVR** | **TAVR** | **SAVR** |
| **No. of patients** | | 77 | 74 | 496 | 454 | 730 | 684 | 145 | 135 |
| **Mean effective orifice area of aortic valve (cm2)** | **Baseline** | 0.67 | 0.74 | 0.8 | 0.8 | 0.8 | 0.8 | 0.7 | 0.7 |
|  | **End of study** | 1.5 | 1.4 | 1.9 | 1.8 | 2.1 | 2.0 | 1.66 | 1.23 |
| **Mean gradient (mmHg)** | **Baseline** | 47.0 | 49.0 | 49.4 | 48.3 | 47.0 | 46.6 | 43.4 | 44.9 |
|  | **End of study** | 13 | 13 | 12.8 | 11.7 | 9.8 | 12.1 | 8.2 | 13.7 |
| **≥ Moderate PVR** | **End of study** | - | - | 30/331 | 0/283 | 2/496 | 0/426 | 6/80 | 0/80 |
| **Bioprosthetic valve failure** | **End of study** | - | - | 3.8% | 3.3% | - | - | - | - |
| **Average KCCQ scores** | **Baseline** | 64 | 65 | 70.3 | 70.1 | 68.6 | 69.4 | - | - |
|  | **End of study** | 80 | 83 | 86.2 | 85.9 | 89.7 | 90.1 | - | - |
| **NYHA Class ≤2** | **Baseline** | 54/77  (70.1%) | 50/74  (67.6%) | 340/495 (68.7%) | 346/453 (76.4%) | - | - | 74/144 (51.4%) | 73/134 (54.2%) |
|  | **End of study** | 49/55  (89.1%) | 39/47  (83%) | 330/391 (84.4%) | 288/335 (86.0%) | - | - | 89.3% | 92% |

**Table S5:** Echocardiographic and functional characteristics at baseline and end of the studies. Abbreviations: KCCQ, Kansas City Cardiomyopathy Questionnaire; NYHA, New York Heart Association; PVR, paravalvular regurgitation; TAVR, transcatheter aortic valve replacement; SAVR, surgical aortic valve replacement.

**Figure S2:** Forest plot comparing transcatheter aortic valve replacement (TAVR) vs. surgical aortic valve replacement (SAVR) for new onset atrial fibrillation and permanent pacemaker (PPM) placement
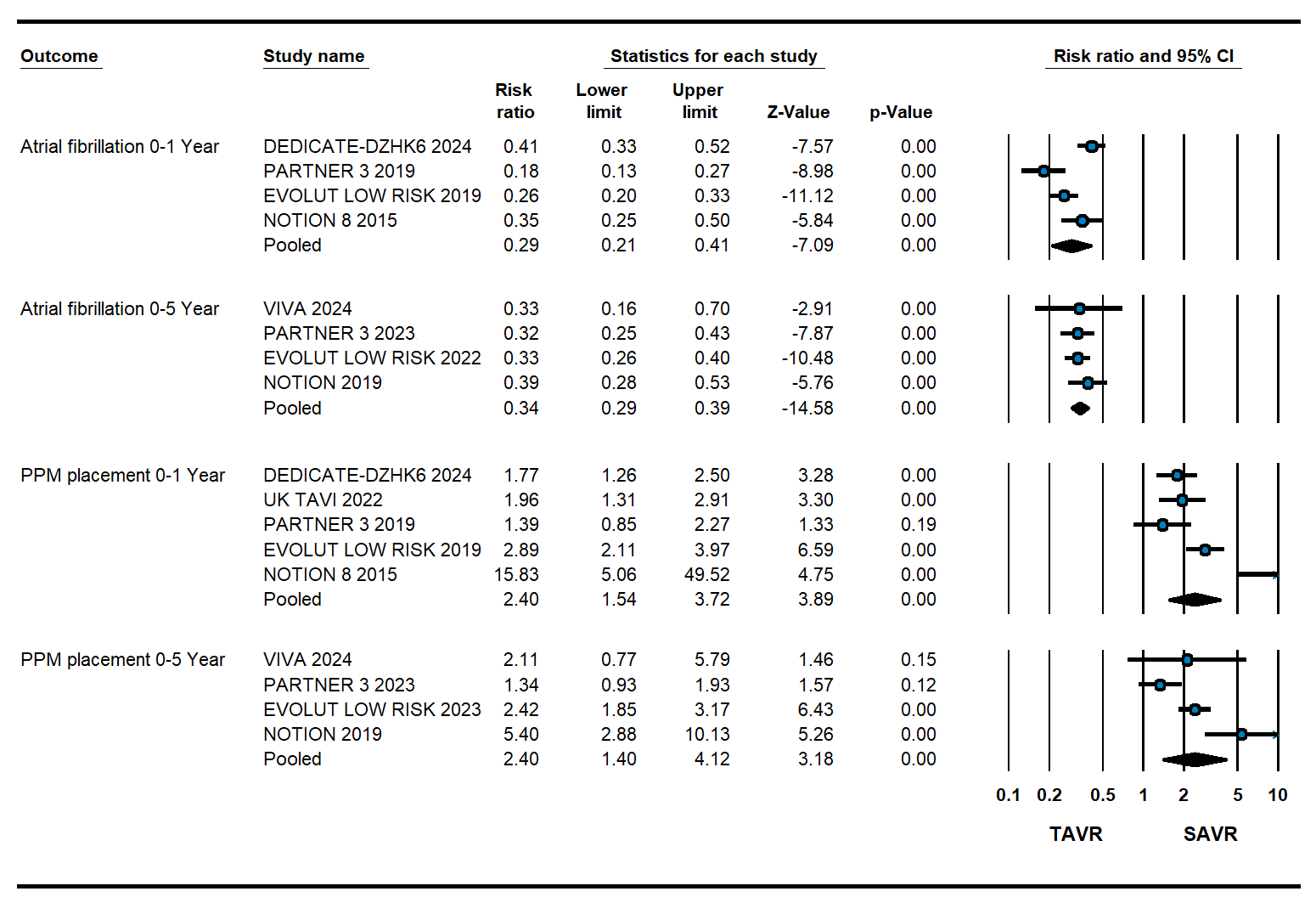


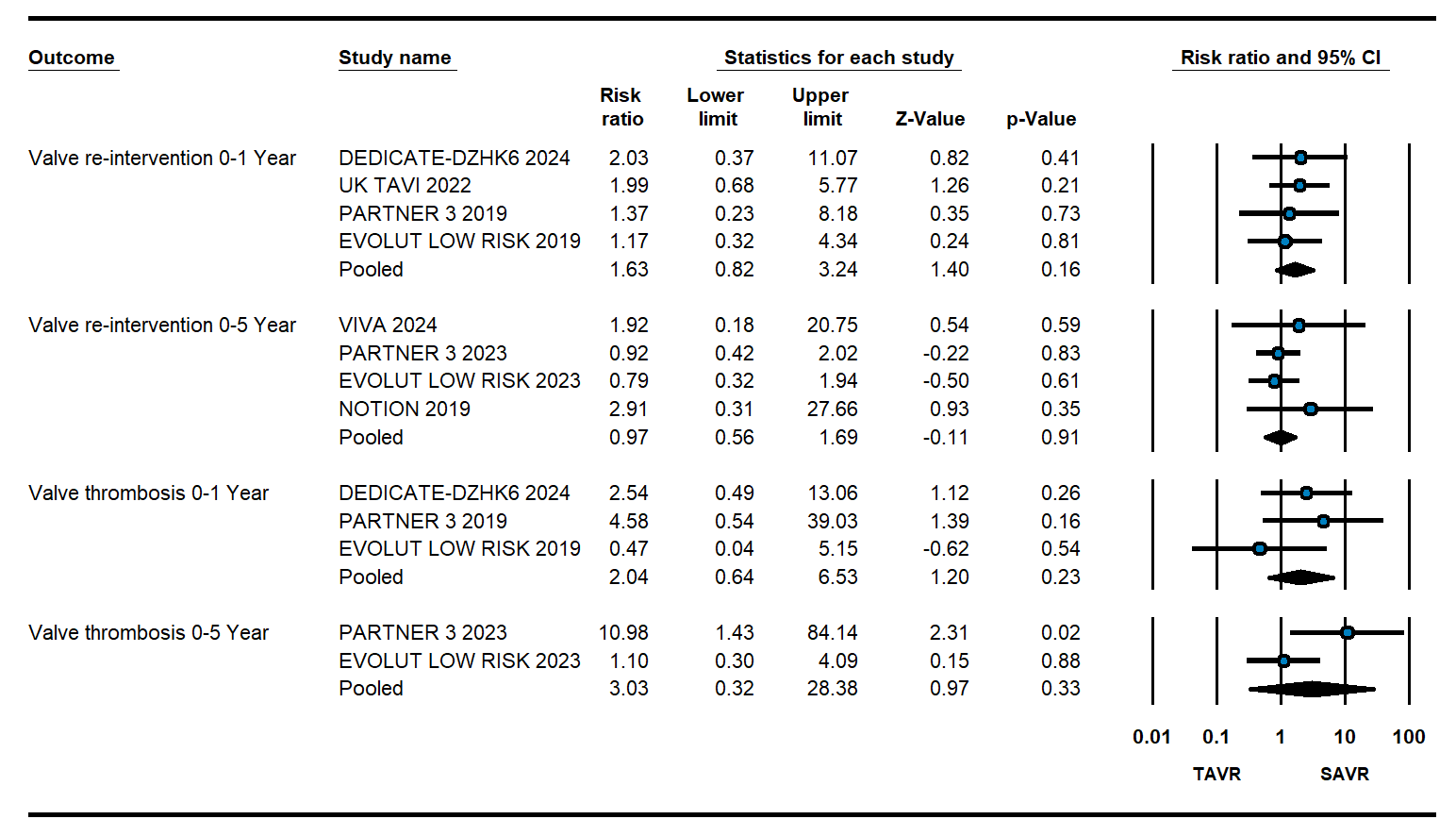
**Figure S3:** Forest plot comparing transcatheter aortic valve replacement (TAVR) vs. surgical aortic valve replacement (SAVR) for aortic valve re-intervention and valve thrombosis.

**Figure S4**: Forest plot comparing transcatheter aortic valve replacement (TAVR) vs. surgical aortic valve replacement (SAVR) for aortic valve area (cm2) and mean gradient (mmHg) by the end of study period.


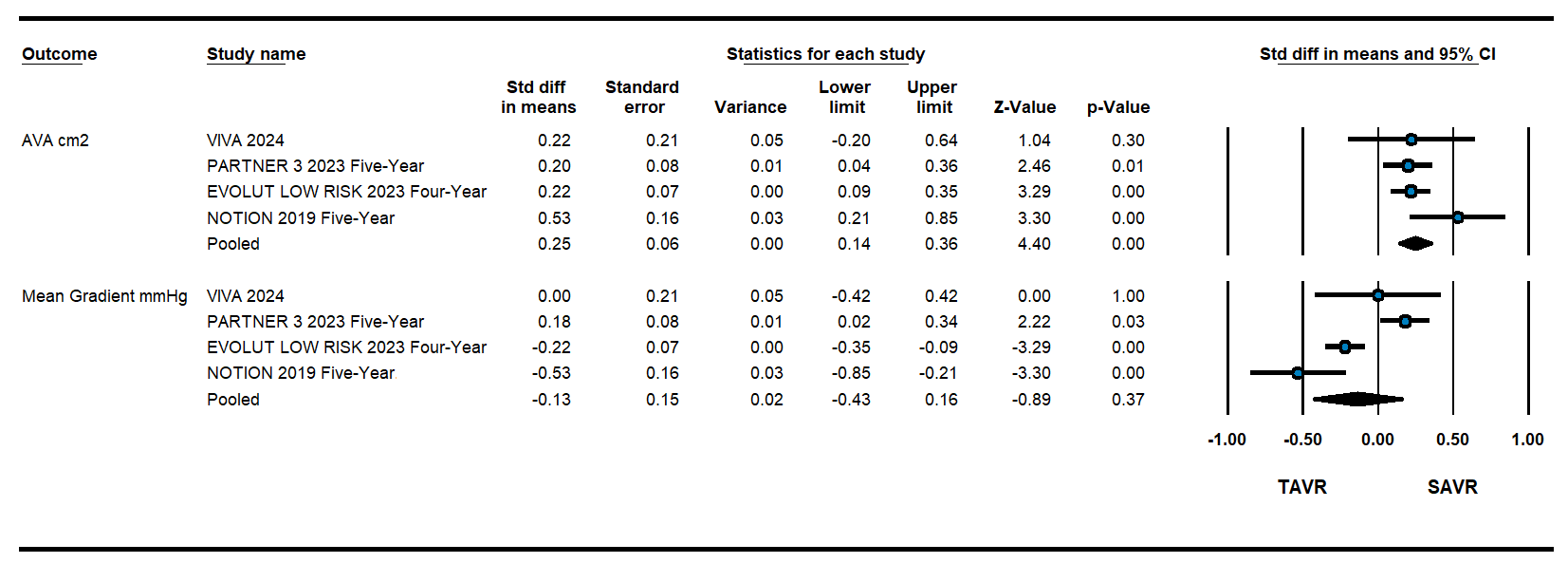


**Figure S5:** Forest plot comparing transcatheter aortic valve replacement (TAVR) vs. surgical aortic valve replacement (SAVR) for moderate or severe paravalvular aortic regurgitation (PVR) by the end of study period.


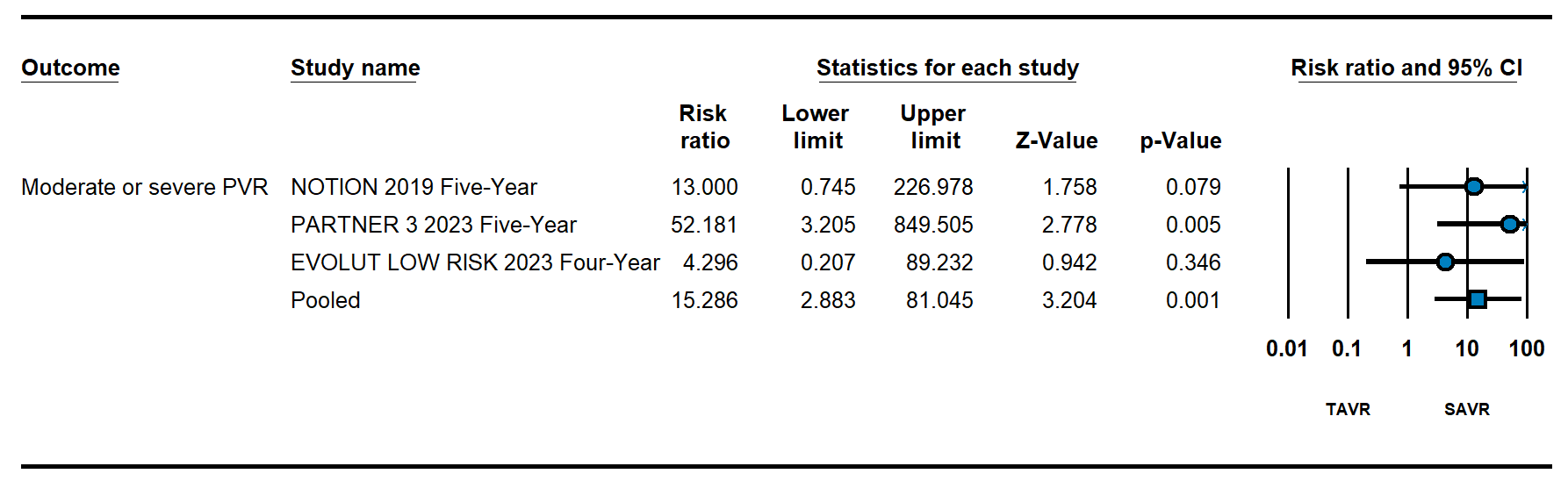

Supplement: Supplementary Data 1 [file mmc1.docx]
